# The influence of resonant leadership on teachers’ innovative behavior: The mediating mechanism of information processing models

**DOI:** 10.1371/journal.pone.0321763

**Published:** 2025-04-11

**Authors:** Yuangen Bao

**Affiliations:** Research Management Center, Shenzhen Longgang District Education Science Research Institute, Shenzhen, China; Alexandria University Faculty of Nursing, EGYPT

## Abstract

Teacher innovation is crucial for adapting education to contemporary societal needs. While the benefits of resonant leadership have been extensively studied, its influence on teachers’ innovative behavior and associated cognitive-affective processes remains underexplored. This study employs the information processing theory to investigate the influence of resonant leadership on innovative behavior among primary and junior teachers in mainland China, while examining the intricate mediating roles of perceived insider status and affective commitment. Using structural equation modeling and bootstrapping techniques, data analysis was conducted on a sample comprising 4,769 teachers from 16 provinces in China. The findings confirm a positive correlation between resonant leadership and teachers’ innovative behavior. Perceived insider status and affective commitment not only independently mediate this relationship but also act as sequential and interactive mediators. Furthermore, the distinct mediating effect of interactive mediation is the most important. This research enhances our understanding of resonant leadership’s impact on basic education while providing novel insights into fostering teacher innovation through comprehensive cognitive-affective pathway analysis.

## 1. Introduction

The development of innovative behavior is pivotal for organizational success in today’s technologically dynamic and globally connected world[1]. Within education, teachers’ innovative behavior is equally critical; as the system’s backbone, they play an irreplaceable role in navigating challenges from technological shifts and globalization [2]. By demonstrating adaptability through innovation, educators ensure institutions remain responsive to evolving demands [3], making the cultivation of such behavior essential for sustained educational advancement.

Leadership styles are widely recognized as key drivers of teacher innovation. Studies demonstrate the impacts of transformational [4], distributed [5], and empowering leadership [6] on teachers’ innovative behavior. However, existing research predominantly examines unidirectional effects, overlooking the reciprocal emotional resonance between leaders and teachers.

Resonant leadership, conceptualized by Goleman [7], counters organizational dissonance by fostering empathetic leader-subordinate relationships [8]. This involves leaders transmitting positive emotions and values to subordinates while also encouraging subordinates to express their emotions and perceptions, facilitating an interactive two-way transmission of emotions. Resonant leaders provide emotional support through empathy, compassion, and mindfulness to help combat stress and burnout among their subordinates [9]. They also identify opportunities within challenges and inspire hope amidst fear and despair for both their organizations and communities [8]. Nevertheless, how resonant leadership specifically drives teacher innovation remains underexplored.

Prior mechanistic studies have relied on isolated frameworks like social cognitive [10], social exchange theory [4], or self-determination theory [6]. Yet integrated analyses of cognitive-affective interactions in teacher innovation remain scarce, prompting calls for multidimensional approaches[11].

Information processing theory suggests organizational contexts activate cognitive-affective systems to shape behavior [12]. The leader behavior style in an organization is a key situational factor that impacts employee cognition, emotion, and ultimately their behavior. We thus propose a dual-path model where resonant leadership drives innovation via cognitive (insider status perception) and affective (emotional commitment) pathways.

Furthermore, based on information processing theory, individual cognitive units can influence behavior by sequentially activating or concurrently interacting with affective units [13]. Moreover, research demonstrates that a high-quality interactive relationship between employees who perceive a strong insider identity and the organization results in higher levels of emotional commitment [14]. Consequently, resonant leadership can impact the interaction between teachers’ perceived insider status and affective commitment, ultimately influencing their innovative behavior. This study also investigates how resonant leadership affects teachers’ innovative behavior through both chain mediation and interactive mediation models.

## 2. Literature review and hypotheses

### 2.1 Information processing model

The information processing model serves as a guiding principle or framework for evaluating the decision-making process of behavioral conduct and constitutes a crucial theoretical foundation for elucidating teachers’ innovative decision-making behavior. This theory has evolved through three key mechanisms, elucidating the evolving roles of affect within cognitive frameworks. According to traditional perspectives, emotions are believed to emerge after cognitive representations. Sensory inputs are encoded and interpreted cognitively before triggering emotional reactions [15]. This suggests that emotions are secondary and depend on pre-existing cognitive structures, emphasizing the primacy of cognition in information processing. For instance, a teacher might first think about the potential benefits and drawbacks of a new method before feeling enthusiastic or anxious about it.

Challenging linear models, Berlyne, et al [16] and Estes, et al [17] proposed that emotions can operate independently from cognitive processing. Zajonc further emphasized the direct influence of emotions on behavior as a distinct pathway in human response systems [18]. For example, a teacher might feel a strong sense of excitement (emotion) that directly motivates them to try a new method, without needing to think through all the details first (cognition). Building upon these foundations, Mischel, et al integrated emotion into social-cognitive processing through their CAPS theory[12]. They proposed Cognitive-Affective Units (CAUs) within personality systems, suggesting that cognitive and emotional units dynamically interact within an individual’s psychological architecture to shape choices and actions collectively. Metcalfe, et al enriched the CAPS model by introducing ‘hot’ (emotion-driven) and ‘cold’ (cognition-driven) systems, providing a nuanced understanding of control dynamics[13]. For example, a ‘hot’ system might drive a teacher to act on a gut feeling to try a new method, while a ‘cold’ system might involve careful planning and analysis before making a decision. Their addition illustrates the bidirectional nature of cognitive-affective interactions guiding human behavior. For instance, a teacher might start with a gut feeling (emotion) to try a new method, then use logical reasoning (cognition) to plan it out, and finally, the excitement (emotion) of seeing positive results reinforces the decision.

In summary, based on the research process of information processing mode in existing literature, the impact of external information on individual behavior primarily occurs through distinct pathways: cognitive system, affective system, cognitive-affective system, and cognitive-affective interaction system. Consequently, within the context of resonant leadership, this study posits that teachers’ decision-making regarding innovative behavior is influenced by their internal cognitive and emotional systems as well as these interrelated systems w1hich operate successively and synchronously; importantly, these four systems are autonomous from one another.

### 2.2 Resonant leadership and teachers’ innovative behavior

In recent decades, there has been an increasing interest in educational leadership because of the growing responsibilities of school principals [19]. The emerging leadership theory of Resonant Leadership was proposed by Western scholars [7]. Goleman and his colleagues believe that leaders, being at the core of a team or organization, are observed and emulated by employees, making them susceptible to negative states that lead to discord in management. Consequently, leaders need to achieve emotional resonance with their employees, conveying positive emotions and adapting to subordinates’ emotions through empathy, making them feel understood and cared for, thus leading to voluntary fellowship. Boyatzis, et al further pointed out that resonant leadership is based on emotional intelligence, encompassing three dimensions: mindfulness, hope, and compassion, which can inspire positive emotions and healthy relationships [20]. Scholars have defined resonant leadership from various perspectives: for instance, Mckee, et al believe that resonant leaders are in sync with those around them [21], Squires and others define resonant leaders as those with high emotional intelligence, and Bawafaa, et al point out that resonant leadership is a positive relational leadership style that empowers team members by instilling confidence and trust [22]. Integrating these perspectives, the essence of resonant leadership lies in three key dimensions: the resonance process between leaders and their team and environment, the manifestation of high emotional intelligence, and the goal of achieving harmony. Leaders should maintain consistency with those around them and the environment, demonstrate individual characteristics such as mindfulness, hope, and compassion, and achieve a harmonious state with themselves, others, and the environment through emotional resonance with their subordinates.

Resonant Leadership, a unique form of leadership, shares similarities with Relational and Emotional Leadership but also exhibits distinct differences. While all three emphasize emotional intelligence in leadership, Resonant Leadership particularly highlights the emotional resonance and deep connections between leaders and team members. Compared to Relational Leadership, which values interpersonal relationships within the team, Resonant Leadership achieves deeper emotional and psychological synchronization with employees through resonance [20]. This synchronization promotes individual harmony as well as harmonious teamwork and organizational state. In contrast to Emotional Leadership’s focus on influencing subordinates’ psychological states through emotional transmission for goal achievement, Resonant Leadership places greater emphasis on emotional resonance and connection between leaders and subordinates, achieving team harmony through mutual adjustment of attitudes and behaviors [23]. Therefore, Resonant Leadership brings positive impacts and long-term development to teams and organizations by stimulating a broader and more profound resonance effect.

Although resonant leadership plays a crucial role in reducing employee anxiety and promoting organizational development, its research is still in the early stages within academic circles [22,24], with limited empirical studies available. This study will focus on the impact of resonant leadership on the innovative behavior of primary and secondary school teachers in mainland China.

Firstly, resonant leaders can achieve the same frequency resonance with employees by transferring emotional energy, to convey their optimism, enthusiasm, and other positive emotions to employees [7]. According to the Extension-Construction principle, teachers with positive emotions such as optimism and enthusiasm can promote their innovative behavior [25]. Secondly, resonant leaders exhibit high levels of emotional intelligence and can manage their emotions effectively and in tune with those of those around them, building strong, trusting relationships and creating an optimistic atmosphere that inspires commitment [24]. This kind of relationship and atmosphere can stimulate the intrinsic motivation of teachers to carry out innovative teaching. Thirdly, resonant leadership is based on emotional intelligence and includes the three dimensions of Mindfulness, Hope, and Compassion, which are the sources of personal renewal [20]. These can not only stimulate teachers’ positive emotions and healthy interpersonal relationships, but also allow teachers to maintain resilience and work efficiently in the face of innovation failures. Therefore, this study proposes the following hypothesis.

H1: Resonant leadership is positively related to teacher innovative behavior.

### 2.3 Mediating effect of teacher perceived inside status

The early conventional information processing model suggests that when exposed to external stimuli, an individual goes through sensory input, followed by physical and advanced encoding of the information, ultimately leading to the formation of cognitive representation. This type of cognitive representation, known as a “cold system” can directly influence an individual’s final judgment and decision-making process without eliciting emotional reactions [13,15,18]. As an important cognitive factor, insider identity perception may play a conductive role between resonant leadership and teachers’ innovative behavior.

The perception of insider identity refers to the self-perception that an individual, as a member of an organization, has won personal space, status, and acceptance in the organization, that is, the degree to which an individual can perceive himself as an “insider” in a particular organization [26]. Leaders are “important others” of employees and possess certain authority [27]. Therefore, employees tend to judge their status in the organization and the extent to which they are accepted by the organization according to the way they treat themselves [28]. When employees perceive the support of their leaders, they tend to judge themselves as “insiders” of the organization [29]. Resonant leadership achieves emotional resonance with teachers by transmitting emotional energy, thereby conveying its own positive emotions such as optimism and enthusiasm to the teachers. This emotional support may foster the teachers’ perception of their sense of belonging. Resonant leadership is when leaders demonstrate a high level of emotional intelligence and can effectively manage their emotions and align with those of those around them, thereby building strong, trusting relationships [24]. This close relationship between leaders and members is an important reason for promoting the perception of employees’ insider status [30].

At the same time, employees’ insider identity cognition also has a significant impact on their work attitude and behavior [26]. Specifically, this study speculated that insider identity cognition would positively affect teacher innovative behavior. According to the social exchange theory, when employees with a high level of insider identity cognition realize the recognition and respect of the organization and meet their needs for emotional connection and belonging, employees will take the initiative to act in a way that is beneficial to the interests of the organization and are more willing to make efforts beyond the work requirements as a return to the organization and positive feedback to the recognition and respect of the organization. In fact, previous studies have shown that employees with a high level of insider identity recognition are more likely to implement innovative behaviors that contribute to the interests of the organization. Based on the above analysis, the following hypothesis is proposed.

H2a: Resonant leadership is positively related to teacher perceived inside status.

H2b: Teacher perceived inside status is positively related to teachers’ innovative behavior.

H2: Teacher perceived inside status play a mediating role between resonant leadership and teacher innovative behavior.

### 2.4 Mediating effect of teacher affective commitment

According to the information processing model, the emotional system can react to external stimuli without intricate reasoning and analysis, functioning as a “thermal system” that directly elicits behavioral responses [12,18]. Innovation process is challenging and risky for the employees [31], so it requires not only the knowledge and skills of employees, but also the intrinsic motivation and emotional support of employees. Affective commitment, the core part of organizational commitment [32], provides a powerful perspective for explaining the mechanisms between resonant leadership and teacher innovative behavior. On one hand, resonant leadership serves as an important information source for shaping teacher affective commitment. Resonant leadership is particularly effective in fostering employees’ sense of organizational emotional support [33], which is rooted in affective commitment [34]. Squires et al. argue that employees are more likely to engage in high quality LMX when they perceive a relationship with their leaders who demonstrate a high level of emotional intelligence [24]. Numerous studies have provided empirical evidence of the positive relationship between LMX and subordinate affective commitment [35].

On the other hand, teacher affective commitment to the school can stimulate their innovative teaching behavior. The extension-construction principle suggests that positive emotions expand employees’ cognition and action, promoting them to generate innovative ideas and adopt creative thinking for problem-solving [25]. Teachers who deeply care about the school closely align their own development with the school goals, prioritizing organizational objectives over personal interests. Such emotionally committed teachers strive to exceed expectations in their work and contribute to the organization’s growth. When conventional methods fail to achieve desired outcomes, these passionate teachers proactively seek new ways and approaches that better suit the organization through active learning. Although there is limited empirical research on the effect of teacher affective commitment on innovative behavior, it is possible to promote innovation based on the above theoretical analysis.

H3a: Resonant leadership is positively related to teacher affective commitment.

H3b: Teacher affective commitment is positively related to teachers’ innovative behavior.

H3: Teachers’ affective commitment plays a mediating role between resonant leadership and teacher innovative behavior.

### 2.5 Chain-mediating role between perceived inside status and affective commitment

Early information processing theory suggests that individual cognitive units activated by situational stimuli can eventually act on individual behavior by activating affective units [15]. Therefore, the relationship between teacher insider perception of identity and affective commitment is not completely independent. As a kind of teacher’s perception of teacher-school relationship, insider perception of identity should positively affect teacher affective commitment. Specifically, employees with a higher level of insider identity perception are recognized and accepted by the organization, have a strong sense of belonging and responsibility to the organization, and show a higher level of affective commitment [36]. Relevant empirical studies also show that when employees have a strong perception level of insider identity, they will incorporate the concept of “in-group” members into their self-concept, forming the overall self-cognition, and thus generating higher emotional commitment [37]. To sum up, this paper constructs a chain mediation model of “resonant leadership-internal personal perception-affective commitment-teacher innovative behavior “. Therefore, this paper makes the following assumptions:

H4: Perceived inside status and affective commitment play a chain-mediating role between resonant leadership and teacher innovative behavior.

### 2.6 Interactive mediating effect of perceived inside status and affective commitment

According to Mischel (1995) theory of cognitive-affective system, the cognitive-emotion unit (CAUs) in personality system will have interactive influence after being stimulated by external environment information, and then determine people’s decision-making and behavior [12]. Therefore, it can be speculated that after primary and secondary school teachers experience resonant leadership behaviors, their internal cognitive factors (insider identity) and emotional factors (affective commitment) may interact and jointly promote their innovative behaviors.

The perception of insider identity refers to an employee’s sense of belonging to the organization, including their cognitive and emotional relationship with it [38]. This relationship also forms the basis for employees’ affective commitment. Therefore, employees’ cognition and emotions towards their organization are internal elements that are closely related to both insider identity perception and affective commitment. In other words, both concepts focus on employees’ cognitive and affective connection with their work organization, suggesting that neither insider identity nor affective commitment alone can fully maximize the effects of employees’ psychological cognition and emotion (e.g., stimulating innovative behavior). They complement each other and together have a more powerful effect than either one alone. Therefore, exploring employee innovative behavior should consider not only the individual impact of perceived inside status and affective commitment but also their interaction. Based on this logic, I expect that:

H5a: Resonant leadership is positively related to interaction between perceived inside status and affective commitment.

H5b: Interaction between perceived inside status and affective commitment is positively related to teachers’ innovative behavior.

H5: The interaction between perceived inside status and affective commitment plays a mediating role between resonant leadership and teacher innovative behavior.

**Fig 1:**
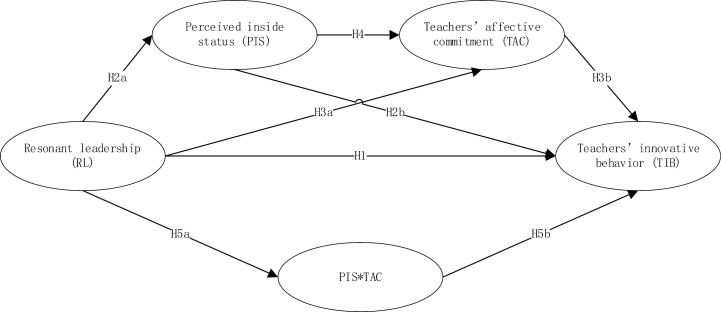
The hypothesized theoretical model.3. Methodology.

Based on the above analysis, the proposed hypothesized theoretical model is illustrated in Fig 1.

### 3.1 Study context and sampling strategy

This study targeted primary and junior high school teachers across mainland China, where regional disparities in educational resources and leadership practices provide a critical context for examining innovation dynamics. We employed stratified random sampling to ensure representation from 16 provinces (50% of China’s 32 provincial units), covering eastern (high-resource urban areas), central (transitional zones), and western (under-resourced rural regions) areas.

### 3.2 Data collection and validity control

The data collection process spanned 14 weeks (February 5 to May 13, 2024) through a self-administered electronic questionnaire distributed via provincial education bureaus’ official platforms, structured into three phases. During the initial two-week preparation phase, we developed the questionnaire using Sojump and conducted a pilot test with 120 teachers to refine clarity and reliability (Cronbach’s α > 0.80 for all scales), collaborating with provincial authorities to secure permissions and coordinate logistics. Subsequently, over eight weeks, invitations were systematically emailed to 5,328 teachers across 16 Chinese provinces (50% of mainland China’s administrative regions) using stratified sampling to ensure regional representation (eastern, central, western). Participants accessed surveys through unique links requiring approximately 15 minutes to complete, with biweekly reminders and ethical safeguards including digital consent forms and anonymized randomized IDs. To ensure data quality, we implemented dual validity checks: automated detection via Sojump filtered 312 responses with duplicates/blank patterns, while manual review eliminated 247 responses exhibiting patterned answers (e.g., straight-lining), aligning with predefined criteria (≤50% missing items, ≤90% response duplication via Qualtrics XM’s fraud detection). This rigorous process yielded 4,769 valid submissions (89.5% validity rate).

### 3. Measures

The study used a five-point Likert scale (ranging from 1 to 5) to measure resonant leadership, teachers’ innovative behavior, perceived inside status, and affective commitment. The scales for resonant leadership, affective commitment, and perceived inside status were translated into Chinese and then back-translated into English by three bilingual graduate students [39]. In this section, we analyzed the reliability and validity of the four questionnaires. For confirmatory factor analysis (CFA), we adopted the model fit criteria recommended by Wen, et al: chi-square model fit criterion (χ^2^)/degree of freedom(DF)≤3, comparative fit index (CFI≥0.90), standardized root mean square residual (SRMR≤0.08), and root mean square error of approximation (RMSEA≤0.08) [40].

#### Resonant leadership.

The scale used to measure principal resonant leadership comprised 10 items divided into four dimensions [41]: (a) self-awareness, (b) social consciousness, (c) self-management, and (d) relationship management. The scale demonstrated high internal consistency with a coefficient α of 0.91. The CFA yielded satisfactory results, indicating good validity: χ^2^/DF =1.93, RMSEA= 0.07, CFI= 0.99, SRMR= 0.02, and RMSEA=0.06.

#### Teachers’ innovative behavior.

We assessed teachers’ innovative behavior using a 23-item scale adapted from Li [42]. This scale, developed within the Chinese context, demonstrated strong reliability and validity. It encompassed two dimensions: innovative idea generation, innovative idea implementation. The scale exhibited high internal consistency, with a coefficient α of 0.88. The CFA yielded satisfactory results, indicating good validity: χ^2^/DF = 1.79, RMSEA= 0.01, CFI= 0.89, and SRMR= 0.02.

#### Affective commitment.

In this study, we employed the affective commitment scale developed by Meyer, et al [43]. The scale comprises 5 items, with higher scores indicating a greater prevalence of affective commitment among teachers. The internal consistency of the scale in our study was strong, with a coefficient α of 0.90.

#### Perceived inside status.

In this study, we employed the perceived inside status scale developed by Stamper, et al [26]. The scale comprises 6 items, with higher scores indicating a greater prevalence of perceived inside status among teachers. The internal consistency of the scale in our study was strong, with a coefficient α of 0.839.

#### Control variables.

Previous research found that demographic characteristics, such as teachers’ gender, education, tenure, can influence teachers’ innovative behavior [44]. To ensure a comprehensive analysis, this study treated gender (as a binary variable), tenure (as a nominal variable), and education level (also as a nominal variable) as control variables. Dummy variables were created for each of these factors, with “female” “≤ 3” and “Undergraduate” serving as the reference groups respectively.

### 3. 4 Ethics statement

This study adheres to the principles outlined in the Declaration of Helsinki and obtained approval from the Human Research Ethics Committee of Longgang Institute of Education Sciences, Shenzhen (Approval No.: 202312280039). All participants, who were K-12 teachers, provided written informed consent before completing the survey. The consent forms outlined the purpose of the study, the rights of the participants, the confidentiality of the data, and how the data would be used. All consent forms are on file and were witnessed by an independent third party. Participants had the option to withdraw from the study at any time. Additionally, our data underwent rigorous anonymization procedures to ensure the utmost participant privacy.

### 3.5 Data analysis

Descriptive statistics, including numbers and percentage distributions, were utilized to characterize the demographic features. For measurement data that followed a normal distribution, the mean ± standard deviation [M (SD)] was employed. Common method bias was assessed via Harman’s single-factor test [45], with a threshold of 40% variance explained. Confirmatory factor analysis (CFA) was conducted to evaluate discriminant validity. Pearson correlation analysis was applied for normally distributed variables, while Spearman correlation analysis was used otherwise. Structural equation modeling (SEM) was employed to test hypothesized relationships among resonant leadership (RL), teachers’ affective commitment (TAC), perceived inside status (PIS), and innovative behavior (TIB) using AMOS 28.0. PROCESS plug-in mediation effect analysis [46] was used to test mediation effects, with statistical significance set at p<0.05 (two-tailed).

## 4. Results

### 4.1 Participant Demographics

The final sample comprised 4,769 teachers. Most respondents came from eastern China (52.02%), while others were from central China (29.89%) and western China (19.09%). These proportions reflect the population distribution across different regions in China. The sample predominantly comprised female teachers (n=3,923) compared to male teachers (n=846), mirroring the gender distribution among primary school educators nationwide. Most teachers possessed at least a bachelor’s degree (86.4%). More than half of the participants had teaching experience exceeding ten years (52.2%).

### 4.2 Preliminary analysis

Before initiating the analysis of structural equation modeling (SEM), it is imperative to consider several data-related issues.

Firstly, the issue of common method biases (CMBs) often arises when utilizing questionnaires for data collection. To address CMB, this study implemented rigorous procedural controls during the data collection process. Furthermore, Harman’s single-factor test was conducted as a means of validation [45]. The results of the unroasted exploratory factor analysis revealed the extraction of 8 factors with eigenvalues exceeding 1.0. However, it is noteworthy that the maximum factor accounted for only 31.69% of the variance, falling below the established threshold of 40%. Therefore, it can be concluded that CMBs did not exert a significant confounding influence on the empirical findings obtained in this study.

Secondly, a confirmatory factor analysis (CFA) was conducted to ensure the distinctiveness of the four variables in this study. The model fit was evaluated using the indices recommended by Wen et al. [40]. As shown in Table 1, the baseline model (four-factor model) demonstrated a satisfactory fit for the four-factor structure (χ^2^/DF=1.73, RMSEA=0.03, CFI=0.91, IFI=0.94, NFI=0.95), outperforming alternative models and indicating good discriminant validity.

**Table 1:** Results of Confirmatory Factor Analysis.

MODLE	FACTOR	χ^2^/df	RMSEA	CFI	IFI	NFI
four-factor	RL; TAC; PIS; TIB	1.73	0.03	0.91	0.94	0.95
three-factor	RL; TAC; PIS+TIB	3.86	0.21	0.95	0.90	0.94
two-factor	RL; TAC+PIS+TIB	8.95	0.08	0.91	0.87	0.86
one-factor	RL+TAC+PIS+TIB	11.35	0.17	0.82	0.79	0.77

Note: RL = resonant leadership, PIS = perceived inside status, TAC = teachers’ affective commitment, TIB = teacher’s innovative behavior, CFI = comparative fit index, IFI = incremental fit index, TLI = Tucker–Lewis index, RMSEA = root mean square error of approximation. The same below.

Furthermore, the sample size in this study met the minimum requirement (n=200) for SEM as suggested by Hu et al. [47] and Kline [48]. We used a probability–probability (PP) plot to assess our data’s conformity to a normal distribution. The detruded PP plot showed that the deviations in our data samples for the four variables were within the range of −0.15 to 0.05, indicating their basic conformity to the normal distribution.

### 4.3 Hypotheses testing

The means and standard deviations of the four main variables in this study are presented in Table 2, along with their respective correlations. These correlation coefficients provide empirical support for further hypothesis testing.

**Table 2:** Descriptive Statistical Analysis of Variables.

Variables	M	SD	1	2	3	4
1.RL	3.19	0.42	1			
2.TAC	3.04	0.59	0.48***	1		
3.PIS	2.88	0.28	0.52***	0.48***	1	
4.TIB	3.36	0.33	0.44**	0.47**	0.64***	1

Note: **, *** respectively represents p<0.01, p<0.001(two-tailed); M: mean; SD: standard deviation.

To test the hypotheses, SEM and bootstrapping tests were conducted following Preacher et al. [49] recommendations. The standardized effect sizes produced by these estimates were comparable to those produced by SEM. The bootstrapping test involved calculating the indirect effects of the independent variables using resampling estimation technique to generate confidence intervals (CIs). In this test, point estimates of total, indirect, and direct effects represented the mean of a bootstrap sample of 5,000 [50].

The SEM model fitting indices for the impact of resonant leadership on teachers’ innovative behavior, with perceived internal status and affective commitment as mediating variables, were χ^2^=1793.86, RMSEA= 0.06, CFI= 0.93, SRMR= 0.04. These indices indicate that the model meets the criteria and is suitable for further interpretation based on recommended values.

The results indicate a significant positive correlation between resonant leadership and teacher’s innovative behavior (γ= 0.39, p<0.01), supporting H1. Resonant leadership also correlates positively with perceived inside status (γ= 0.49, p<0.001), supporting H2a. Perceived inside status is significantly correlated with teacher’s innovative behavior (γ= 0.58, p<0.001), supporting H2b.Resonant leadership further shows a significant positive correlation with affective commitment (γ=0.42, p<0.001), providing support for H3a. Affective commitment is significantly correlated with teacher’s innovative behavior (γ= 0.44, p < 0.001), supporting H3b.

In order to examine the mediating role of resonant leadership in teachers’ innovative behavior, we utilized the mediation analysis method proposed by Wen et al. [40]. The Process program was used for analysis, specifically Model 4 and Model 6, to determine the specific mediating effects of each variable. Bootstrap resampling with 5000 samples was employed, while a confidence interval of 95% was set. The combined analysis results using model4 and model6 are shown in Table 3.

**Table 3:** Mediation Analysis Results.

Path		Effect	Bootstrapping				
**SE**	**t**	**p**	**LLCI**	**ULCI**
**Total effect**		0.52	0.12	5.89	0.000	0.01	0.06
**Direct effect**		0.21	0.07	4.27	0.000	0.16	0.48
**Indirect effects**	Total effect	0.31	0.01			0.04	0.09
	Indirect 1: PIS	0.07	0.02			0.02	0.05
	Indirect 2: TAC	0.09	0.03			0.01	0.04
	Indirect 3: PIS*TAC	0.12	0.01			0.03	0.08
	Indirect 4: PIS→TAC	0.03	0.02			0.01	0.14
	Indirect 1 minus Indirect 2	-0.02	0.01			0.01	0.036
	Indirect 1 minus Indirect 3	-0.05	0.07			0.11	0.16
	Indirect 1 minus Indirect 4	0.04	0.10			0.02	0.13
	Indirect 2 minus Indirect 3	-0.03	0.09			0.16	0.19
	Indirect 2 minus Indirect 4	0.06	0.08			0.03	0.11
	Indirect 3 minus Indirect 4	0.09	0.06			0.07	0.19

The confidence interval for the main effect of resonant leadership on teachers’ innovative behavior does not include 0, indicating a significant impact. Hypothesis H1 is supported once again. When considering intermediary variables such as perceived inside status, affective commitment, and their interaction terms, the confidence interval for the total indirect effect also excludes 0, suggesting a significant indirect effect. Specifically, the indirect effect 1 had a significant effect value of 0.07, as the bias-corrected 95% CI interval did not include 0, confirming the significance of H2. The indirect effect 2 also showed significance with an effect value of 0.09 and a bias-corrected 95% CI interval that did not include 0, confirming H3. Similarly, the indirect effect 3 had an effect value of 0.12 and a bias-corrected 95% CI interval that did not include zero, supporting the significance of H5. Lastly, the indirect effect 4 had an effect value of only 0.03 but still demonstrated significance as its bias-corrected confidence interval excluded zero. Furthermore, based on difference testing and standardization coefficients associated with each mediator variable, it can be concluded that the interaction between perceived inside status and affective commitment exerts the most substantial influence.

## 5. Conclusion and discussion

### 5.1 Interpretation of the ﬁndings

Our study uncovered a robust positive relationship between resonant leadership and teachers’ innovative behavior, indicating that the demonstration of resonant leadership by principals significantly promotes and nurtures such behavior. This finding is consistent with relevant research conducted in diverse cultural contexts [51,52], thereby enhancing the credibility and reliability of the findings. By fostering a supportive and emotionally engaging environment, resonant leaders can directly encourage teachers to adopt more innovative practices, contributing to a more dynamic and adaptive educational setting. This direct effect highlights the critical role of leadership in driving innovation within schools, emphasizing the potential of resonant leadership as a powerful tool for educational transformation.

We have identified a mechanism by which resonant leadership influences teachers’ innovative behavior. The perception of internal status and affective commitment independently mediate the relationship between resonant leadership and teachers’ innovative behavior, playing both a chain-mediated and interactive-mediated role. Collectively, these four mediated paths account for 59.61% of the total impact effect with the interactive mediation being the most significant. This finding aligns with the predictions and theories of information processing [12]. Unlike much of the existing literature, which primarily focuses on the personality and psychological characteristics of employees, our study delves into the psychological processes, specifically the interaction between cognitive and emotional factors. By applying the information processing theory, we provide a deeper understanding of how resonant leadership influences teachers’ innovative behavior. This research not only advances the systematic study of leadership’s impact on employee innovation but also extends the application of information processing theory to the field of education, enriching its scope and practical relevance.

The innovative finding of this study highlights the significant mediating role of the synchronic interaction between insider identity and affective commitment, which is the most impactful among the four mediating paths, accounting for 38.71% of the total effect. This finding is crucial as it deepens our understanding of the mechanisms through which resonant leadership influences teachers’ innovative behavior within the school context. Our results provide robust empirical support for the information processing theoretical framework proposed by Mischel, et al. [12], which posits that cognitive and emotional units dynamically interact within an individual’s psychological structure, jointly influencing decision-making and behaviors. The synchronic interaction between insider identity and affective commitment reveals a unique and powerful pathway, underscoring the importance of both cognitive and emotional processes in shaping teachers’ innovative behavior. These findings contribute to the broader literature on leadership and innovation by demonstrating the critical role of a holistic cognitive-affective approach in fostering a supportive and innovative educational environment. Resonant leaders, through their empathetic and emotionally intelligent leadership, create a context that leverages both cognitive and emotional factors, leading to increased teacher innovation and organizational success.

### 5.2 Practical implications

Firstly, our study establishes a solid foundation for principals’ self-improvement and development, enabling them to engage in reflective practices regarding their resonant leadership and strive for continuous improvement. Through an analysis of the four empathic ways in which leaders’ cognition and emotion interact, this paper validates the positive impact of resonant leadership on enhancing teachers’ innovative behavior. Consequently, education administration departments could implement graded and classified resonant training programs, such as monthly reflection sessions and role-playing exercises, to standardize the process and provide comprehensive content guidance. This will foster school leaders who possess both exceptional talent and emotional intelligence, thereby better serving the innovative development of teachers’ profession. In promoting principals, attention could be given not only to their management skills but also to their emotional intelligence and proficiency in handling organizational conflicts.

Secondly, our study provides valuable insights for principals on how to foster teachers’ innovative behavior. Principals could prioritize enhancing teachers’ internal identity perception and affective commitment, as well as motivating them to engage in innovative practices. In the context of China’s relationship-oriented culture, teachers place great importance on their connection with the school. The notion of being an “insider” within the school community can stimulate teachers’ sense of responsibility towards the institution, strengthen their identification and emotional attachment to it, and encourage behaviors that benefit the organization. For example, principals can organize regular team-building activities, such as workshops and social events, and set up a teacher lounge with amenities like coffee machines and comfortable seating. On one hand, schools can enhance recruitment, training, promotion, reward and punishment mechanisms by implementing appropriate and effective human resource management practices. They can also create a cooperative, open-minded, harmonious, and inclusive environment that supports teacher development while fostering their internal identity perception and emotional attachment to the school. On the other hand, principals could attend to both material and spiritual needs of teachers by continuously improving their well-being. Recognizing and valuing teachers’ contributions through annual awards and public recognition can enhance their sense of belongingness and attachment to the school.

Finally, our study highlights the significance of synchronic interaction between insider identity and affective commitment as a crucial link between resonant leadership and teachers’ innovative behavior. Therefore, in terms of school management, principals need to strengthen the internal governance structure of schools, encourage teachers to participate in decision-making and school management, and adopt various effective measures to comprehensively cultivate teachers’ perception of insider identity and affective commitment to the school. For example, principals can establish a Teacher Advisory Board to involve teachers in key decisions, hold regular school hall meetings to gather feedback, and provide opportunities for teachers to lead professional development sessions.

### 5.3 Limitations and future research

While this study provides valuable insights into the mechanisms through which resonant leadership influences teachers’ innovative behavior, several limitations should be acknowledged. First, the sample was drawn from a single country, which may limit the generalizability of the findings. Cultural factors, such as the degree of collectivism versus individualism, can significantly influence the effectiveness of leadership styles and the nature of workplace relationships. For example, in collectivist cultures, where group harmony and interdependence are highly valued, the impact of resonant leadership on teachers’ sense of insider identity and affective commitment might be more pronounced. Conversely, in individualistic cultures, where personal achievement and independence are emphasized, the effects of resonant leadership might manifest differently. To address this limitation, future research should include schools in different countries and cultural contexts. This would provide a broader perspective and allow for a more comprehensive understanding of how resonant leadership operates in various educational environments. Additionally, future studies could explore the specific cultural dimensions that moderate the relationship between resonant leadership and teachers’ innovative behavior, such as power distance, uncertainty avoidance, and long-term orientation. By examining these cross-cultural differences, researchers can develop more nuanced and culturally sensitive strategies for fostering innovation in schools.

Furthermore, this study relied on self-reported data, which may be subject to response biases. Future research could incorporate multiple data sources, such as peer evaluations and observational data, to provide a more robust and multi-faceted understanding of the phenomena under investigation. Finally, longitudinal designs could be employed to examine the long-term effects of resonant leadership on teachers’ innovative behavior and to track changes over time.
